# Parvovirus B19-Induced Membranoproliferative Glomerulonephritis in an Immunocompetent Adult Patient: A Case Report

**DOI:** 10.7759/cureus.87038

**Published:** 2025-06-30

**Authors:** Arij Ouanjine, Fatma Fendri, Remy Kerdraon, Manon Dekeyser

**Affiliations:** 1 Department of Nephrology, Centre Hospitalier Universitaire d'Orléans, Orléans, FRA; 2 Department of Pathology, Centre Hospitalier Universitaire d'Orléans, Orléans, FRA; 3 Orléans Interdisciplinary Laboratory for Innovation and Research in Health, Université d'Orléans, Orléans, FRA

**Keywords:** intravenous immunoglobulin, lupus-like, membranoproliferative glomerulonephritis, parvovirus-b19, rituximab

## Abstract

A 41-year-old woman without significant medical history was admitted for edema and a 10 kg weight gain. Two months earlier, she had experienced a flu-like syndrome treated with amoxicillin. At admission, she presented with severe hypertension and stage 1 acute kidney injury. Work-up revealed nephrotic syndrome, microscopic hematuria, and transient biological hemolysis. Type II cryoglobulinemia was identified, along with complement consumption. Autoimmune and viral serologies were negative. Renal biopsy revealed a full-house membranoproliferative glomerulonephritis (MPGN). She was initially treated as having type II cryoglobulinemic MPGN with rituximab, corticosteroids, and nephroprotection. Subsequently, an acute coexisting parvovirus B19 infection was confirmed by seropositivity for IgG and IgM and high viremia. This was associated with an inflammatory articular flare. Rituximab was stopped and replaced by intravenous immunoglobulin (IVIg), leading to clinical and renal remission and viral clearance over a 10-month period. Although rare, parvovirus B19 is a known cause of lupus-like MPGN, even in immunocompetent adults. Failure to recognize primary parvovirus B19 infection exposes patients to diagnostic delay and unwarranted treatment. Timely IVIg therapy avoids persistent disease and prevents treatment escalation.

## Introduction

Parvovirus B19 (PVB19) is a non-enveloped, single-stranded DNA virus most commonly associated with erythema infectiosum in children. In adults, PVB19 infection can produce systemic manifestations such as hematologic abnormalities, arthritis, and, more rarely, renal pathology. Renal complications include membranoproliferative glomerulonephritis (MPGN), acute post-infectious glomerulonephritis (GN), focal segmental glomerulosclerosis, and thrombotic microangiopathy [1,2].

These lesions are often immune complex-mediated and can simulate autoimmune glomerulopathies, notably lupus nephritis, owing to similar histologic patterns [3]. Such overlap can lead to misdiagnosis and inappropriate treatment. PVB19 can also act as an infectious trigger for type II cryoglobulinemia and its renal manifestations; targeted antiviral or supportive management is often sufficient, with immunosuppression reserved for severe or refractory cases [2]. Detection of capsid proteins or PVB19 DNA in renal tissue can support the diagnosis, but these tools are not always accessible and may lack sensitivity [4].

While many cases resolve spontaneously in immunocompetent patients, severe systemic presentations may require targeted therapy. Intravenous immunoglobulin (IVIg) has shown efficacy in treating prolonged or severe PVB19-related disease, particularly in cases of persistent viremia [5].

Here, we report a rare case of PVB19-induced MPGN associated with type II cryoglobulinemia in an immunocompetent adult, highlighting the importance of viral screening in atypical GN presentations.

## Case presentation

A 41-year-old woman was admitted to the nephrology department of our hospital for symptomatic anemia and pitting edema, with a weight gain of 10 kg over two weeks. She had no significant past medical history except for active smoking. She had two healthy children and one early miscarriage. Two months prior, she had reported a flu-like illness with high fever, pharyngitis, and myalgia, for which she received amoxicillin for seven days.

On physical examination, the patient exhibited stage II hypertension, lower limb edema, and normal body temperature. There were no signs of arthritis or arthralgia. Laboratory investigations revealed stage 1 acute kidney injury, nephrotic syndrome, and microscopic hematuria. These findings were associated with anemia, thrombocytopenia, undetectable haptoglobin, and elevated markers of hemolysis. A positive direct Coombs test, as well as low complement fractions (C3 and C4), were also noted. Three consecutive tests confirmed the presence of type II cryoglobulins. Viral and bacterial serologies were negative, except for a false-positive Epstein-Barr virus result. Antinuclear antibodies (ANA) were detected, while other autoimmunity markers were negative, notably anti-dsDNA antibodies. Serum protein electrophoresis and immunofixation were normal. A 2-deoxy-2-[^fluorine-18^]fluoro-D-glucose (¹⁸F-FDG) PET/CT scan revealed hypermetabolism of the spleen with both supradiaphragmatic and infradiaphragmatic lymphadenopathy.

**Figure 1:**
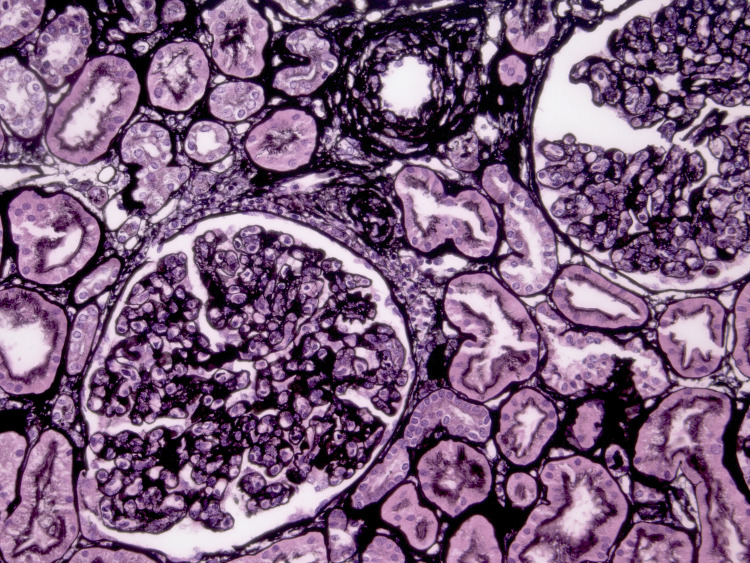
Light Microscopy (Jones Silver Stain) Light microscopy of renal biopsy with Jones Silver stain (original magnification ×200) showing mesangial hypercellularity, lobulated glomeruli, and subtle duplication of the glomerular basement membrane. Tubular structures are intact. These are characteristic features of membranoproliferative glomerulonephritis

**Figure 2:**
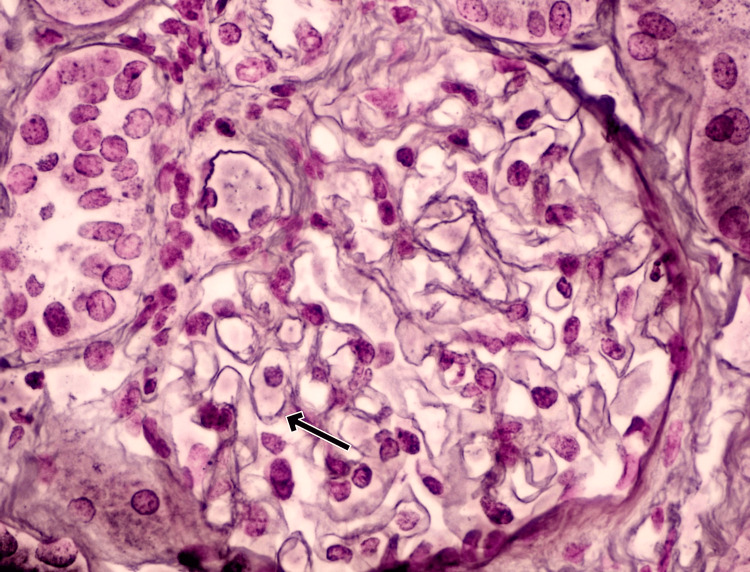
Light Microscopy (Reticulin Stain) Light microscopy of renal biopsy with reticulin stain (original magnification ×400) shows closer glomerular lobulation and more prominent duplication of the glomerular basement membrane (arrow indicates double contours), consistent with membranoproliferative glomerulonephritis pattern.

**Figure 3:**
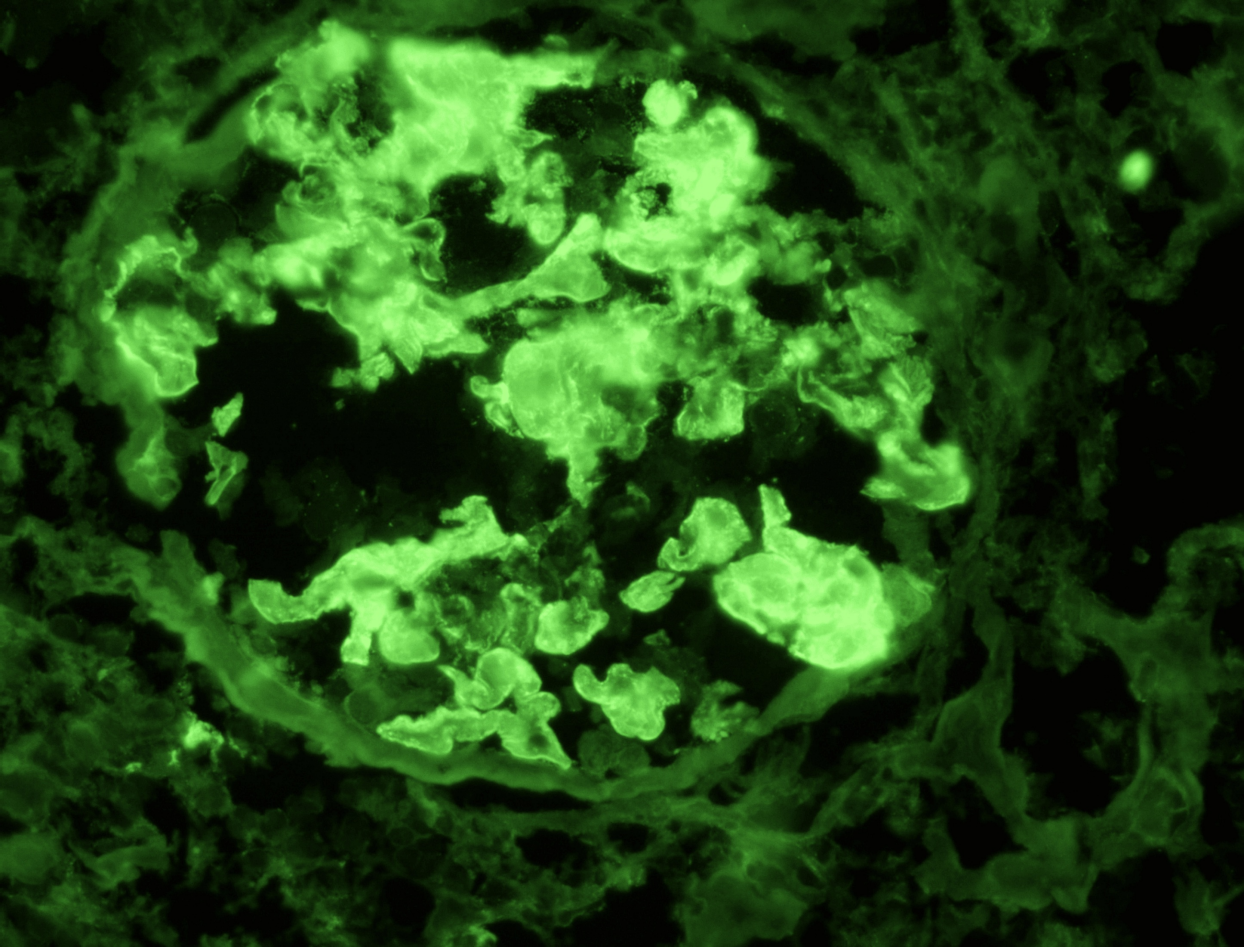
IgG Immunofluorescence Direct immunofluorescence reveals intense granular immunoglobulin G deposition along the glomerular capillary loops, supporting an immune complex-mediated glomerular injury.

**Figure 4:**
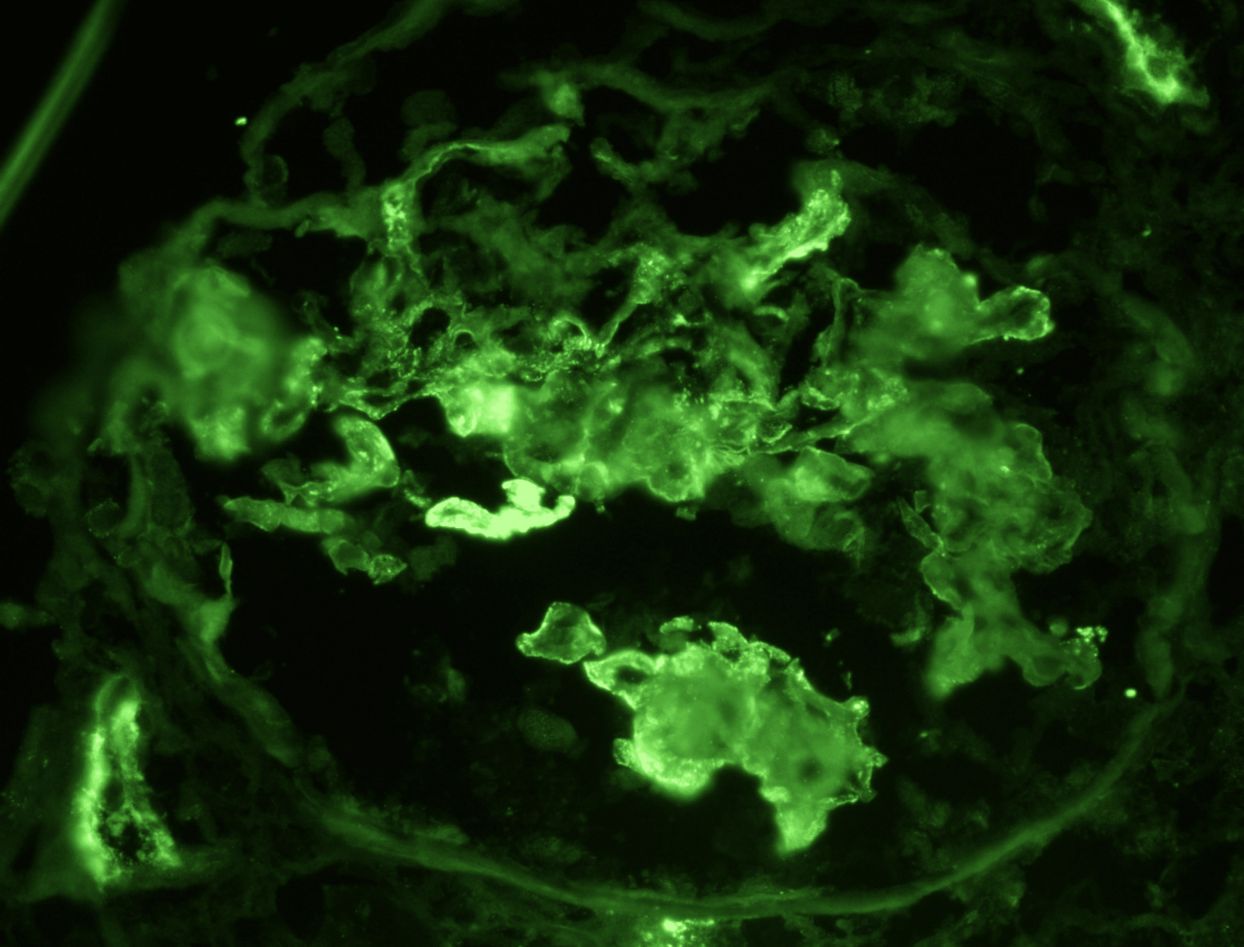
IgA Immunofluorescence Weak to moderate immunoglobulin A staining is seen in mesangial and peripheral capillary areas, compatible with polytypic immune complex deposition, though not dominant.

**Figure 5:**
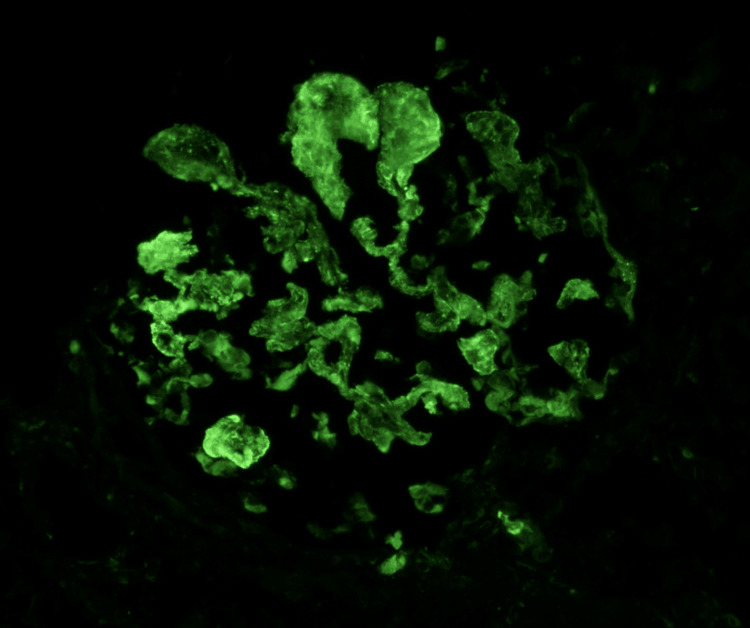
C1q Immunofluorescence Granular capillary wall deposition of C1q (complement component 1q) with weak intensity is observed, consistent with activation of the classical complement pathway, a characteristic feature of immune complex–mediated glomerulonephritis.

**Figure 6:**
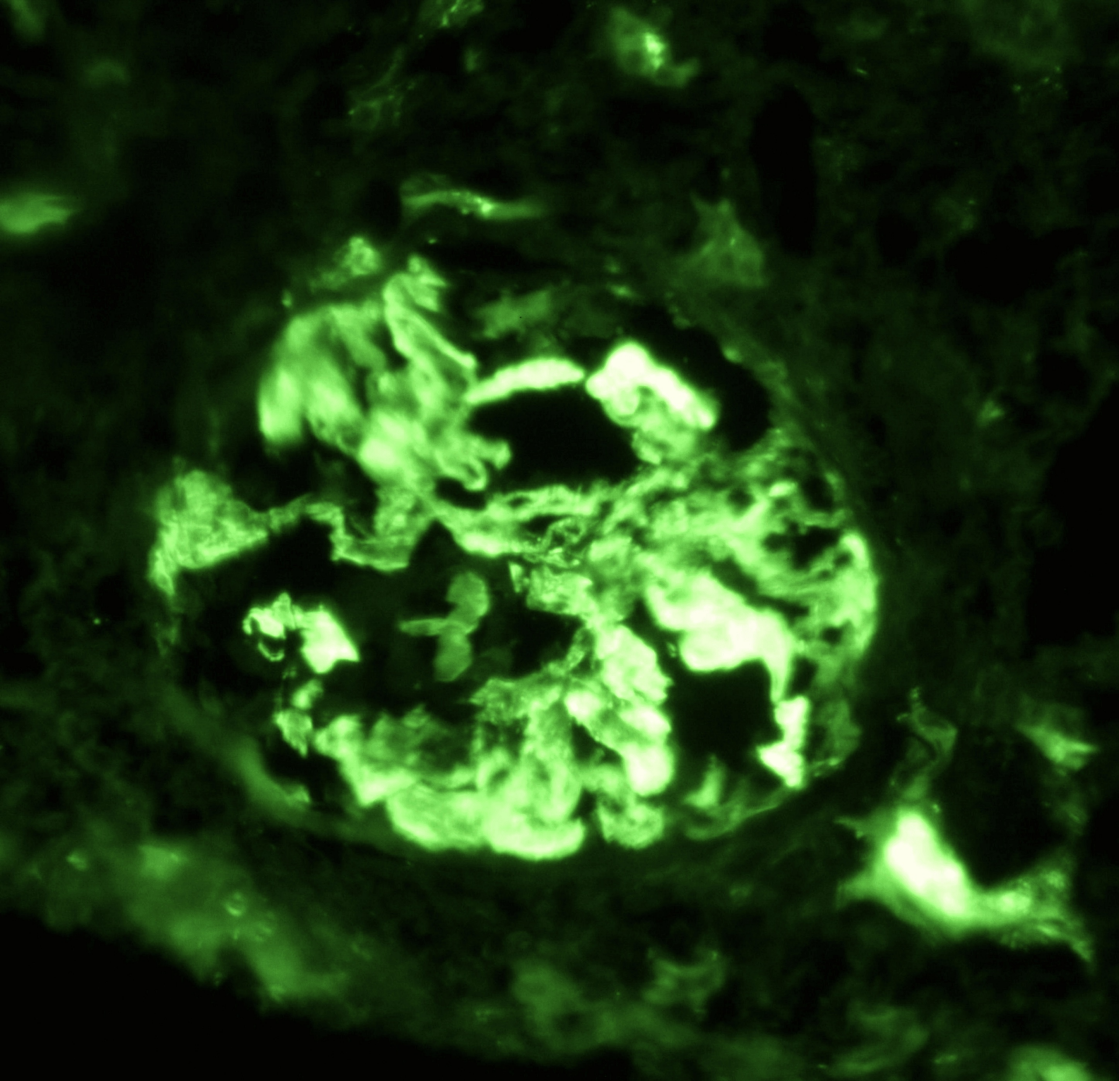
C3 Immunofluorescence Immunofluorescence highlights strong, coarse C3 (complement component 3) staining along the capillary loops, suggestive of classical or alternative complement pathway activation.

A renal biopsy was performed. The cortex contained about 20 glomeruli, all pathological, suggesting an MPGN (Figure 1) with full-house immunofluorescence (immunoglobulin (Ig) G, IgA, C1q, C3) (Figures 2-6), consistent with a lupus-like pattern. No signs of crescent formation or extracapillary proliferation were observed.

A diagnosis of type II cryoglobulinemic MPGN was established, with no neurological damage, no arthritis, and no patent infectious or autoimmune disease. Moreover, there were not enough criteria for a systemic lupus erythematosus (SLE) diagnosis. At this point, a rituximab-based therapy was initiated, along with progressively tapered oral corticosteroids and an angiotensin-converting enzyme (ACE) inhibitor. No plasma exchange was performed.

One month later, she developed a symmetrical polyarthritis affecting the small joints of the hands, wrists, knees, and feet. At that point, delayed serology results confirmed acute PVB19 infection, with both IgM and IgG positivity and high viremia, while the patient still presented with stage 1 acute kidney injury, persistent nephrotic syndrome, and microscopic hematuria. We performed additional diagnostic tests on the kidney biopsy. PVB19 DNA testing on the renal tissue could not be performed because the sample was insufficient, while the immunohistochemical staining for PVB19 (antibodies B19, clone R92F6 of VP1/VP2 capsid protein) was negative. Rituximab was discontinued, and the patient was treated with intravenous immunoglobulin (IVIg), resulting in clinical improvement.

Ten months later, the patient achieved complete clinical and renal remission; serum creatinine normalized, blood pressure was controlled, and nephrotic-range proteinuria resolved, allowing discontinuation of antihypertensive therapy.

Laboratory data trends are summarized in Table 1.

**Table 1:** Summary of Laboratory Results UPCR, urine protein-to-creatinine ratio; WBC, white blood cells; RBC, red blood cells; CRP, C-reactive protein; LDH, lactate dehydrogenase; ESR, erythrocyte sedimentation rate; DAT, direct antiglobulin test IgG; ANA, antinuclear antibodies; APSA, antiphospholipid syndrome antibodies; ANCA, anti-neutrophil cytoplasmic antibodies; MPO, myeloperoxidase; PR3, proteinase 3; GBM, glomerular basement membrane; PLA2R, phospholipase A2 receptor; CCP, cyclic citrullinated peptide; PCR, polymerase chain reaction; VCA, viral capsid antigen; EBNA, Epstein–Barr nuclear antigen; EBV, Epstein–Barr virus; HIV, human immunodeficiency virus; HBs, hepatitis B surface antigen; HBc, hepatitis B core antibody; HCV, hepatitis C virus; HSV, herpes simplex virus; VZV, varicella zoster virus; CMV, cytomegalovirus; TSH, thyroid-stimulating hormone; PTH, intact parathyroid hormone

Parameters	Patient Values at Admission	Patient Values at One Month	Patient Values at 10 Months	Unit	Reference Range
Serum Creatinine	107	87	72	µmol/L	45–90
Serum Albumin	29	23	37	g/L	35–50
UPCR	7.5	6.6	<0.10	g/g	<0.2
Urine WBC	15	11	<5	/mm³	<10
Urine RBC	229	158	8	/mm³	<10
Hemoglobin	11.4	13.6	12.2	g/dL	12.0–16.0
Platelet Count	199	205	192	×10⁹/L	150–450
Haptoglobin	Undetectable	0.33	-	g/L	0.3–2.0
LDH	373	396	-	U/L	<250
CRP	2.1	0.2	0.7	mg/L	<5
ESR (1h/2h)	10/20	-	-	mm	<20/<40
DAT IgG	Positive	-	Negative	-	Negative
C3	0.48	-	1.38	g/L	0.81–1.57
C4	0.07	-	0.28	g/L	0.13–0.39
Cryoglobulin	Type II Cryoglobulinemia (positive)	Type II Cryoglobulinemia (positive)	Negative	-	Negative
ANA	Positive (1:160)	Positive (1:180)	-	-	Negative
Anti-DNA (native)	Negative	-	-	-	Negative
Anti-SSA / Anti-SSB	Negative	-	-	-	Negative
Anti-Sm / Anti-RNP	Negative	-	-	-	Negative
APSA	Negative	-	-	-	Negative
Anti-MPO / Anti-PR3	Negative	-	-	-	Negative
Anti-GBM	Negative	-	-	-	Negative
Anti-PLA2R	Negative	-	-	-	Negative
Anti-CCP	-	Negative	-	-	Negative
Rheumatoid Factor (IgM)	-	Negative	-	-	Negative
IgG Anti-Parvovirus B19	10.76	8.14	2.10	U/mL	<0.90
IgM Anti-Parvovirus B19	18.24	12.72	4.79	U/mL	<0.90
PCR Parvovirus B19	6.45	5.65	Negative	log₁₀ copies/mL	<0.1
IgM Anti-VCA	26,8	-	-	U/mL	<20
IgG Anti-VCA	476,5	-	-	U/mL	<20
IgG Anti-EBNA	224	-	-	U/mL	<5
PCR EBV	<1.57	-	-	log₁₀ copies/mL	<1,3
HIV 1/2 Antigen/Antibodies	Negative	-	-	-	Negative
Syphilis serology	Negative	-	-	-	Negative
HBs Antigen (HBV)	Negative	-	-	-	Negative
Anti-HBs	68	-	-	-	≥10 U/L
Anti-HBc	Negative	-	-	-	Negative
Anti-HCV	Negative	-	-	-	Negative
IgG Anti-HSV	346,4	-	-	-	<0,9 UI/mL
IgG Anti-VZV	132,5	-	-	-	<0,9 UI/mL
IgG Anti-CMV	<6,0	-	-	-	<6,0 UI/mL
IgM Anti-CMV	<0,9	-	-	-	<0,9 UI/mL
25 OH Vitamin D	28.9	-	52.4	µg/L	20–50
TSH	0.579	-	0.50	mIU/L	0.350–4.940
PTH 1-84	24.3	-	18.8	ng/L	5.5–38.4

## Discussion

Different types of PVB19-associated kidney injuries have been reported. Most cases describe immune-complex-mediated GN, including classic acute post-infectious GN, membranoproliferative patterns, membranous nephropathy, and infection-related IgA nephropathy, often presenting with abrupt nephrotic syndrome and acute kidney injury. Our patient presented with severe lupus-like MPGN associated with type II cryoglobulinemia, which fits into the most commonly described category. Less frequently, podocytopathies such as collapsing focal segmental glomerulosclerosis (FSGS), conventional FSGS, and minimal change disease are reported. Rare cases of vascular lesions, such as thrombotic microangiopathy, have been observed in both native and allograft kidneys [1,2].

P-antigen, also called globoside or Gb4, is the cell-surface glycosphingolipid that PVB19 uses as its primary receptor. PVB19 initiates renal injury by binding P-antigen on glomerular endothelial and epithelial cells, permitting direct cytopathic entry. Viral replication sparks interferon-α and pro-inflammatory cytokine release, recruiting neutrophils and monocytes and exposing neo-antigens that trigger classical complement activation [2]. Circulating viral immune complexes then settle in the mesangium and capillary loops, intensifying complement-mediated damage, stimulating mesangial proliferation and matrix expansion, and producing a membranoproliferative pattern [3]. Resultant endothelial dysfunction with platelet activation may progress to thrombotic microangiopathy, whereas tubules largely escape direct infection but can sustain secondary hypoxic stress. Altogether, glomerular involvement in PVB19 nephropathy arises from the synergy of direct viral cytotoxicity, immune-complex deposition, and complement activation [2]. In most immunocompetent patients, viral replication ceases within weeks, circulating immune complexes dissipate, and the deposited glomerular immune complexes are resorbed, leading to the spontaneous recovery of renal function [5].

In our case, we could not provide formal histological proof of direct renal injury. The current diagnostic triad includes immunohistochemistry (IHC) for capsid proteins (VP1/VP2), in situ hybridization (ISH) for viral DNA, and tissue polymerase chain reaction (PCR). IHC staining for PVB19 is highly specific but not highly sensitive; VP1/VP2 IHC shows 66% sensitivity and 100% specificity, while ISH increases sensitivity to 83% without compromising specificity. Tissue PCR is the most sensitive, with a 90% detection rate. Detection of PVB19 DNA in kidney tissue is considered strong evidence of renal injury causality [3-5]. Nevertheless, in a retrospective cohort of 100 renal biopsies, Kauffmann et al. identified four cases of PVB19-associated nephropathy. All four patients had acute PVB19 infection (both IgM and IgG positive), and tissue PCR, though used as the reference method, was positive in only one case; the timing of kidney injury and the favorable spontaneous renal evolution (except in one patient with chronic PVB19 infection) argued for the causality of PVB19 [1]. Moreover, renal PCR is not always available, and a positive result may reflect past (inactive) infection and must be interpreted alongside current viremia and serology [3]. Therefore, a multimodal diagnostic approach, incorporating both biological and clinical features, is recommended, particularly as treatment depends on confirming active infection.

Regarding therapeutic strategy, our patient received IVIg to treat an acute, hyperalgesic, prolonged, and corticosteroid-resistant polyarthritis that manifested only after rituximab administration. Acute PVB19 infection can manifest with nonspecific polyarthralgias, typically involving small joints symmetrically [6]. In Wolfromm’s series of 82 patients, arthralgia was reported in 47% of immunocompetent individuals but only in 19% of immunodeficient patients [7]. In the context of lupus-like GN, this viral arthritis can easily mimic and mislead to a connective tissue disease diagnosis. Establishing the viral etiology helps avoid unnecessary immunosuppression; in a case reported by Georges et al., discontinuing steroids led to regression of both arthritis and nephritis [8]. Our patient experienced a similar favorable articular and renal outcome only after suspending rituximab and tapering corticosteroids to withdrawal. Most uncomplicated adult cases resolve spontaneously within two to four weeks [6]; IVIg is reserved for prolonged or immunocompromised cases, which correlate with persistent viremia and predict responsiveness to IVIg [9]. Early recognition of viremia and timely IVIg administration can shorten clinical suffering and prevent overtreatment.

PVB19-associated renal injury often follows a self-limited course. Nakazawa et al. reported spontaneous complete renal recovery within eight weeks, suggesting that viral clearance and immune-complex resorption, which precede clinical resolution, can occur without pharmacologic intervention [5]. In the absence of a standardized treatment protocol, therapeutic strategies vary according to the severity of renal tissue injury. When histology reveals crescentic or lupus-like lesions, many groups implement corticosteroids (pulse methylprednisolone followed by tapering oral doses) [8]. Lazzerini et al. combined steroids, rituximab, and plasmapheresis to manage cryoglobulinemic vasculitis and renal injury [10]. These previous successes contrast with our patient’s initial worsening under immunosuppressive therapy.

IVIg therapy is the cornerstone of treatment for persistent or severe PVB19 infection. Kauffmann et al. administered IVIg at 2 g/kg over two days to three adults with anemia and proliferative GN; all showed timely hemoglobin normalization, viremia clearance by day 10, and up to a 50% reduction in proteinuria within one month [1]. After reviewing 118 adult cases, Jacquot et al. reported that renal responses mirrored hematologic ones, with an overall 80% response rate to IVIg at 0.4 g/kg/day for five days [6]. They observed faster improvement when treatment was initiated within four weeks of symptom onset. Wolfromm et al. recommend starting IVIg in immunocompromised or anemic patients, or when organ inflammation is uncontrolled, and giving extra courses only for relapses; no adverse events other than transient hyperviscosity were noted [7].

Recurrent PVB19-associated renal injury is well documented and potentially preventable. One kidney-transplant recipient relapsed three times despite initial IVIg therapy. Subsequently, weekly subcutaneous immunoglobulin (SCIg) at 0.1 g/kg maintained protective IgG levels above 10 g/L, preserved PCR negativity, and prevented relapses over an 18-month follow-up [11]. Antiviral treatments targeting single-stranded DNA viruses are notably absent from the PVB19 management arsenal; ribavirin and cidofovir lack proven efficacy and pose nephrotoxicity risks [12,13].

## Conclusions

Although uncommon, PVB19 infection is a documented trigger of lupus-like MPGN, even in immunocompetent hosts. A symptomatic primary parvovirus infection is notably difficult to recognize. Consequently, patients are prone to a diagnostic odyssey that may delay appropriate treatment, potentially altering or prolonging the disease’s course toward spontaneous resolution. Prompt initiation of IVIg therapy in severe or relapsing injuries is the key to the disease’s management, alongside individualized immunomodulation according to histologic features and immune status.
